# COVID-19-related stigmatization among a sample of Egyptian healthcare workers

**DOI:** 10.1371/journal.pone.0244172

**Published:** 2020-12-18

**Authors:** Aya Mostafa, Walaa Sabry, Nayera S. Mostafa

**Affiliations:** 1 Department of Community, Environmental, and Occupational Medicine, Faculty of Medicine, Ain Shams University, Cairo, Egypt; 2 Institute of Psychiatry, Faculty of Medicine, Ain Shams University, Cairo, Egypt; Chinese Academy of Medical Sciences and Peking Union Medical College, CHINA

## Abstract

**Objectives:**

To explore coronavirus disease 2019 (COVID-19)-related stigma and its associated factors among Egyptian physicians.

**Methods:**

A cross-sectional study using an anonymous online questionnaire was conducted from 7 to 21 June 2020. The survey was distributed via social media and email to physicians working in Egypt through convenience sampling.

**Results:**

509 physicians participated in the study (mean age: 41.5±10.2). 138 (27.1%) participants were directly involved in the care of COVID-19 patients. 159 (31.2%) participants reported severe level of COVID-19-related stigma. Participants’ mean overall COVID-19-related stigma score was 40.6±8.0. The mean subscale scores were: personalized stigma 26.0±5.7, disclosure concerns 9.3±2.2, negative self-image 6.9±1.6, and concern with public attitudes 24.4±4.9. In the multivariable regression analyses, the overall COVID-19-related stigma score was higher in participants with lower qualifications (β = -0.19, 95% CI: 2.32, -0.64, p = 0.001), and in those working in a quarantine hospital (β = 0.08, 95% CI: 0.01, 7.14, p = 0.050).

**Conclusions:**

A considerable proportion of Egyptian physicians in this exploratory study experienced COVID-19-related stigmatization. These preliminary findings highlight the need for specific research and targeted interventions particularly addressing COVID-19-related stigmatization among healthcare workers.

## Introduction

Coronavirus disease 2019 (COVID-19) is a respiratory infection that is recognized as a serious global public health threat [1]. Epidemics provoke social stigma [2], especially when surrounded with several uncertainties, as in the case of the COVID-19 pandemic. Imposing unfamiliar measures to protect public health, improper understanding of modes of transmission, health resource shortages, and conflicting messages from authorities are amongst the many factors that led to public fear and anxiety during the COVID-19 pandemic [3].

As of 1 August 2020, 17,859,763 COVID-19 cases and 685,179 related deaths occurred globally [4]. More than 10% of these global cases were among healthcare workers (HCWs) [5], who are at the frontline of the COVID-19 pandemic response. Besides being occupationally exposed to a higher risk of infection, HCWs reported longer shifts, psychological distress, burnout, and stigma [6]. More than 200 COVID-19-related attacks on the healthcare workforce and facilities occurred, not only in developing (Bangladesh, India, Mexico, and Malawi), but also in developed countries (the USA) [7]. In these incidents, HCWs faced social isolation, public insults or harassment, refusal of public transportation use, and house eviction. Despite these incidents, COVID-19-related stigma and its associated factors among HCWs have not been adequately investigated.

The conceptual framework for the process of health-related stigma constitutes an interplay of drivers and facilitators that influence several outcomes among affected individuals [8], such as the right of healthcare, housing, burial, advocacy, and appreciation from community members [9]. In the case of COVID-19-related stigma among HCWs, drivers were mostly fear of infection, concerns about social judgment, and self-blame or blame to others for being a source of infection or for the consequent adverse outcomes [10]. Facilitators in this case were the availability of occupational safety standards and protective equipment for HCWs in healthcare facilities [11, 12]. ‘Infodemic’ (excessive circulation of misinformation) also acted as a driver or facilitator for COVID-19-related stigmatization [10]. The interplay of these drivers and facilitators resulted in stigma ‘marking’ of HCWs, which manifested as stigma experiences and practices. Stigma experiences included ‘self’, ‘perceived’, ‘anticipated’, ‘associative’, and ‘experienced discrimination’. ‘Self-stigma’ involved internally adopting negative beliefs and feelings of others and social devaluation of the important HCWs’ role in the COVID-19 pandemic [13]. ‘Perceived stigma’ denoted perceptions about how HCWs are treated in a specific context [14]. ‘Anticipated stigma’ manifested as expectations of being perpetrated by others if HCWs’ occupation becomes known [15]. ‘Associative stigma’ was the stigma experienced by HCWs’ family, friends or HCWs who treated COVID-19 patients [16]. ‘Experienced discrimination’ included the stigmatizing behaviours that may or may not be illegal, such as house eviction or verbal abuse [11]. Stigma practices included stereotyping (beliefs associated with being a HCW), prejudice (devaluation of HCWs), stigmatizing behaviour (social avoidance during daily activities, for example, shopping), and discriminatory attitudes (belief that HCWs should be disallowed from full community participation) [11, 17].

COVID-19-related stigma manifestations were reported in several countries, including in Egypt. As of 1 August 2020, Egypt had a total of 94,316 COVID-19 cases and 4,834 related deaths [18]. More than 3,000 physicians were infected and 152 died [19]. The local news reported several incidents of Egyptian HCWs being shunned by others for the fear of being infected [20]. Moreover, a physician who died due to her COVID-19 illness was denied burial by fearful and protesting villagers causing immense psychological distress for her family [21]. This incident put COVID-19-related stigmatization among HCWs under the spotlight; local authorities promptly responded and attempts to show public support for HCWs took place. However, there was a general attitude among authorities to indict HCWs, especially those who got infected, for not being fully committed to their duties and/or the required infection precaution measures.

To our knowledge, research examining the extent of COVID-19-related stigmatization among HCWs using a stigma-specific tool is scarce–on both local and global levels. Measuring the magnitude of stigma and understanding its relevance to HCWs’ direct involvement in COVID-19 care pathways and other associated factors may help the concerned authorities address this issue more effectively. Such research may also provide preliminary evidence to design targeted public health education interventions. In this context, this study explores COVID-19-related stigma and its associated factors among Egyptian physicians.

## Subjects and methods

### Study design and participants

A cross-sectional study using an anonymous online self-administered questionnaire was conducted from 7 to 21 June 2020. Physicians working in Egypt during the COVID-19 pandemic were recruited using convenience sampling. A sample size of 455 physicians was calculated at a 99% confidence level and an alpha error of 5%, based on the assumption that 22% of physicians would report COVID-19 related stigma, a proportion similar to what HCWs had previously reported during the Severe Acute Respiratory Syndrome (SARS) epidemic [22]. Another 10% (n = 45) was added in case of non-response or missing data; the target sample was 500 physicians. Physicians working outside Egypt were excluded from the study. Participants were recruited until the sample size was achieved. There were no other exclusion criteria.

### Study tools and data collection

The online questionnaire was developed using Microsoft forms through the secure platform of the Faculty of Medicine and an official e-mail address that is password-protected and available only to the research team. The link to the questionnaire was distributed through various social networks and emails. Participants were encouraged to share the form with workmates. No incentives were provided for participation. By clicking the link, an introductory page displayed the study rationale, its aims, noting participation was voluntary, ensuring strict anonymity and confidentiality of responses, and showed the informed consent statement. After providing consent, the participant was directed to complete the three sections of the questionnaire (see S1 Questionnaire):

#### Section 1

Personal and occupational data: age, gender, marital status, residence, qualification, specialty, workplace, provision of direct care to COVID-19 patients, and COVID-19 infection status.

#### Section 2

Perceived COVID-19-related stigmatization: Literature search revealed no specific tools or scales to measure COVID-19-related stigma. We widened the search scope to include any tool for measuring stigma in a similar epidemic caused by a respiratory virus. The only available tool was that developed by Verma et al. [17], who adapted the HIV Berger scale [23] to measure stigma among HCWs during the 2004 SARS epidemic in Singapore. Verma et al. stigma scale consisted of 17 items that were categorized into *four subscales*: *personalized stigma; disclosure concerns; negative self-image; and concern with public attitudes*. The four-point Likert scale responses were: “strongly disagree”, “disagree”, “agree”, and “strongly agree”, scoring 1 to 4, respectively. The total or overall COVID-19-related stigma score was calculated as the sum of the scores of its 17 items, ranging from 17 to 68, with a higher score indicating a higher level of stigma. The 17 items of the stigma scale used in the current study that were adapted from Verma et al. had a Cronbach’s alpha of 0.906, suggesting high internal consistency. The four subscales were calculated as the sum of their individual constituting items, noting that individual items can be assigned to more than one subscale (see S1 Questionnaire). The subscale items were: personalized stigma (11 items), disclosure concerns (4 items), negative self-image (3 items), and concern with public attitude about HCWs (10 items) (see S1 Questionnaire).

#### Section 3

This section included questions about:

- facilitators to health-related stigma: role of the media in addressing COVID-19 public stigma against HCWs, and receiving psychological support/counselling to cope with the COVID-19 pandemic;
- stigma experiences by physicians, including:

#### Self-stigma

feeling guilty of exposing HCWs’ family to infection with COVID-19.

#### Anticipated stigma

needing to hide HCWs’ positive COVID-19 result.

#### Associative stigma

staying away from the family till the crisis subsides if the HCW is in regular contact with COVID-9 patients.

#### Experienced discrimination

who were the people most stigmatizing HCWs and attitude towards refusal of burial of dead COVID-19 HCW cases, as such incident occurred in Egypt.

c) physicians’ major concerns during the COVID-19 pandemic and what measures they thought may help them most to cope with the COVID-19 pandemic.

### Validation and pilot study

Face and content validity of the questionnaire were assessed by three experts in occupational medicine, psychiatry and public health. The questionnaire was administered in English, as physicians in Egypt study Medicine in English and are familiar with the language. A pilot study was performed on 10 physicians to assess the clarity and appropriateness of the questions and answer categories, and the feasibility of the study. Feedback from the pilot study was followed by minor rephrasing modifications of the questionnaire items. The first modification was performed to one item of the stigma scale changing the word “touching me” into “getting close to me”. The other adjustment was in the wording of the question about experienced discrimination. The 10 questionnaires from the pilot study were not included in data analysis.

### Ethical considerations

The Faculty of Medicine, Ain Shams University Research Ethics Committee approved the study (FMASU R 22 / 2020). Informed consent was obtained from all participants.

### Statistical analysis

Using IBM SPSS 25.0, descriptive statistics including mean and standard deviation (SD), or median and interquartile range (IQR) were performed to describe continuous data, while frequencies and proportions were calculated to describe categorical variables. Bivariate analysis was done using chi-squared test, independent samples t-test, Analysis of Variance (ANOVA), and linear regression to identify the factors associated with participants’ direct involvement in the care of COVID-19 patients, as well as participants’ overall COVID-19-related stigma scores. As individual items may fall under more than one subscale, the total stigma score is not a simple summation of the four subscale scores. To enable direct comparison of the four subscales, percentiles and 95% confidence intervals (CI) were calculated: participants’ scores for each subscale were divided by the total possible score for the corresponding subscale [24]. Using the calculated percentiles, the overall stigma scores were categorized into three categories: 0–33% = no or mild stigma, >33% and < 66% = moderate stigma, and ≥66% = severe stigma. This approach was suggested by Charles et al., given the lack of universal cut-off points for stigma scores [25]. Multivariable linear regression analyses were performed to determine factors associated with the following outcomes (expressed as continuous scale variables): overall COVID-19-related stigma score, and scores of the: personalized stigma subscale, disclosure concern subscale, negative self-image subscale, and concern with public attitudes about HCWs subscale. The independent variables that showed statistically significant associations with each of these outcomes in the bivariate linear regression analysis were introduced into the multivariable model using the enter method. These included age; gender (male, female); marital status (unmarried, married), qualification (MBBCH, Diploma, Master, Doctorate), residence (Cairo, other governorate), specialty (Internal Medicine, Surgery, other), workplace (quarantine hospital, fever hospital, public hospital, primary healthcare center, university hospital, private hospital/clinic, other), direct involvement in the care of COVID-19 patients (no, yes), current work in a COVID-19 affected healthcare facility (no, yes), having a positive test result (no, yes) if the participant has done a test to check whether they got infected. A p-value of ≤ 0.05 was considered statistically significant.

## Results

### Sample characteristics

The mean age of the 509 participants was 41.5±10.2, ranging from 24 to 70. Most participants were females (n = 353, 69.4%), married (n = 978, 74.3%), had a post-graduate degree (n = 443, 87%), and lived in Cairo (n = 420, 82.5%). More than half of the participants specialized in Internal Medicine (n = 261, 51.3%) and almost a half of the participants worked in a university hospital (n = 242, 47.5%), while less than 1 in 20 of the sample worked in a quarantine hospital for COVID-19 patients (n = 19, 3.7%) or a fever hospital (n = 2, 0.4%) **Table 1**.

**Table 1:** Background characteristics and total COVID-19-related stigma scores among a sample of HCWs, Egypt, 2020 (n = 509).

	Total	Directly involved in the care of COVID-19 patients		Statistic^b^	p-value^c^	Total stigma score	Statistic^d^	p-value^e^
		No	Yes			Mean±SD		
	N = 509	n = 371	n = 138					
**Age group **								
24–34	144 (28.3)	86 (23.2)	58 (42.0)	18.227	<0.001	42.7±6.8	6.124	**<0.001**
35–44	179 (35.2)	136 (36.8)	43 (31.2)			40.6±7.9		
45–54	123 (24.2)	98 (26.5)	25 (18.1)			38.9±8.6		
55+	62 (12.2)	50 (13.5)	12 (8.7)			39.0±9.1		
**Gender, n (%) **								
Male	156 (30.6)	103 (27.8)	53 (38.4)	5.36	0.021	40.2±8.4	0.873	0.452
Female	353 (69.4)	268 (72.2)	85 (61.6)			40.8±7.9		
**Marital status, n (%) **								
Unmarried	131 (25.7)	82 (22.1)	49 (35.5)	9.456	0.002	42.0±7.4	0.854	**0.020**
Married	378 (74.3)	289 (77.9)	89 (64.5)			40.1±8.2		
**Qualification, n (%) **								
MBBch	66 (13.0)	42 (11.3)	24 (17.4)	15.292	0.002	45.3±6.5	12.951	**<0.001**
Diploma	18 (3.5)	14 (3.8)	4 (2.9)			41.5±6.6		
Master	161 (31.6)	104 (28.0)	57 (41.3)			41.3±6.9		
Doctorate	264 (51.9)	211 (56.9)	53 (38.4)			38.9±8.6		
**Governorate of residence, n (%) **								
Cairo	420 (82.5)	66 (17.8)	23 (16.7)	0.088	0.767	40.5±7.9	0.534	0.380
Other governorates	89 (17.5)	305 (82.2)	115 (83.3)			41.3±8.8		
**Specialty, n (%) **								
Internal Medicine	261 (51.3)	171 (46.1)	90 (65.2)			39.7±8.4		
Surgery	115 (22.6)	87 (23.5)	28 (20.3)	17.432	<0.001	40.5±7.1	5.719	**0.003**
Other	133 (26.1)	113 (30.5)	20 (14.5)			42.5±7.7		
**Workplace**^**a**^**, n (%)**								
Quarantine hospital for COVID-19 cases	19 (3.7)	4 (1.1)	15 (10.9)	26.836	<0.001	45.6±7.7	0.968	**0.006**
Fever hospital^**f**^	2 (0.4)	0	2 (1.4)	5.398	0.020	48.0±5.7	0.327	0.191
Public hospital	76 (14.9)	45 (12.1)	31 (22.5)	8.457	0.004	43.1±7.5	1.025	**0.003**
Primary healthcare center	19 (3.7)	14 (3.8)	5 (3.6)	0.006	0.937	42.5±7.8	0.486	0.284
University hospital	242 (47.5)	175 (47.2)	67 (48.6)	0.077	0.842	39.3±8.4	4.791	**<0.001**
Private hospital/clinic	116 (22.8)	78 (21.0)	38 (27.5)	2.424	0.119	40.2±7.7	0.911	0.508
Other	116 (22.8)	104 (28.0)	12 (8.7)	21.374	<0.001	40.9±7.5	1.531	0.687

^a^ More than one option was allowed; numbers do not sum to total

^b^ χ2 statistic for Chi-squared test

^c^ P-values ≤ 0.05 indicate statistically significant differences between participants who were directly involved in the care of COVID-19 patients and those who were not.

^d^ F statistic for Independent Samples t-test or ANOVA

^e^ P-values ≤ 0.05 indicate statistically significant differences of total stigma scores by background characteristics

^**f**^ Fever hospitals in Egypt are the hospitals which receive acute febrile illness patients seeking medical advice. Hence, fever hospitals received COVID-19 suspected and confirmed cases during the epidemic.

Two-fifths of the participants reported they currently worked at a COVID-19 affected healthcare facility during the period of survey administration (n = 219, 43.0%), less than a quarter of whom were involved in triage for COVID-19 case detection (n = 49, 22.9%). Only a quarter of the participants had done tests to check whether they got infected (n = 128, 25.1%); 19 (14.8%) of those indicated they had a positive test result **Table 2**.

**Table 2:** COVID-19-related activities and total COVID-19-related stigma scores among a sample of HCWs, Egypt, 2020 (n = 509).

	Total	Directly involved in the care of COVID-19 patients		Statistic^a^	p-value^b^	Total stigma score	Statistic^c^	p-value^d^
		No	Yes			Mean±SD		
	N = 509	n = 371	n = 138					
**Are you currently working in COVID-19 affected healthcare facility?**								
No	290 (57.0)	254 (68.5)	36 (26.1)	73.685	<0.001	40.4±8.0	0.655	0.519
Yes	219 (43.0)	117 (31.5)	102 (73.9)			40.9±8.1		
**In which care pathway are you involved?**	**N = 219**	n = 117	n = 102					
Triage for COVID-19 case detection	49 (22.4)	10 (8.5)	39 (38.2)	55.501	<0.001	40.6±8.2	0.528	0.663
Intensive care unit	16 (7.3)	2 (1.7)	14 (13.7)			42.1±7.9		
Quarantine rooms for COVID-19 cases	16 (7.3)	5 (4.3)	11 (10.8)			40.6±7.4		
Other	138 (63.0)	100 (85.5)	38 (37.3)			39.8±8.5		
**Have you personally done any tests to check whether you got infected with COVID-19?**								
No	381 (74.9)	308 (83.0)	73 (52.9)	48.479	<0.001	40.3±8.2	0.578	0.142
Yes	128 (25.1)	63 (17.0)	65 (47.1)			41.5±7.5		
**If yes, what was the test?**	**N = 128**	n = 63	n = 65					
PCR	94 (73.4)	42 (66.7)	52 (80.0)	2.916	0.088	40.8±7.5	0.021	0.073
Antibodies	34 (26.6)	21 (33.3)	13 (20.0)			43.5±7.3		
**What was the result?**	**N = 128**	n = 63	n = 65					
Negative	109 (85.2)	57 (90.5)	52 (80.0)	2.778	0.096	41.2±7.3	0.485	0.296
Positive	19 (14.8)	6 (9.5)	13 (20.0)			43.2±8.5		

^a^ χ2 statistic for Chi-squared test

^b^ P-values ≤ 0.05 indicate statistically significant differences between participants who were directly involved in the care of COVID-19 patients and those who were not.

^c^ F statistic for Independent Samples t-test or ANOVA

^d^ P-values ≤ 0.05 indicate statistically significant differences of total stigma scores by background characteristics

Approximately a quarter of the sample reported they were directly involved in the care of COVID-19 patients (n = 138, 27.1%) **Table 2**. In the bivariate analysis, participants who were directly involved in the care of COVID-19 patients were significantly more likely to report being younger, male, unmarried, and specialized in Internal Medicine, working in a quarantine, fever, public hospital, or a COVID-19 affected healthcare facility, and had performed a COVID-19 test–than those who were not directly involved in the care of COVID-19 patients **Tables 1 and 2**.

### COVID-19-related stigma

The mean scores of participants’ overall COVID-19-related stigma scores and the four subscales scores are shown in **Table 3**. The mean overall COVID-19-related stigma score was 40.6±8.0. The mean scores for the subscales were: personalized stigma 26.0±5.7, disclosure concerns 9.3±2.2, negative self-image 6.9±1.6, and concern with public attitude 24.4±4.9 **Table 3**. The score percentiles indicated that the participants perceived higher stigma related to the concern with public attitudes about HCWs subscale (61.0, 95% CI: 60.0, 62.2) and comparatively lower stigma related to the negative self-image subscale (57.5, 95% CI: 56.3, 58.7). Approximately one third of the participants reported a severe level of COVID-19-related stigma (n = 159, 31.2%), 327 (64.2%) reported a moderate level of stigma, and 23 (4.5%) reported no or a mild level of stigma. This pattern was also true for the subscales.

**Table 3:** Total COVID-19-related stigma and subscales scores among a sample of HCWs, Egypt, 2020 (n = 509).

						Directly involved in the care of COVID-19 patients		Statistic^a^	p-value^b^
						No (n = 371)	Yes (n = 138)		
Stigma subscales	Number of items	Total possible score	Mean±SD	95% CI	Percentile (95% CI)	Mean±SD	Mean±SD		
**Personalized stigma**	11	44	26.0±5.7	25.5, 26.5	59.1 (57.9, 60.2)	25.9±5.8	26.1±5.7	0.004	0.630
**Disclosure concerns**	4	16	9.3±2.2	9.1, 9.5	58.1 (57.2, 59.6)	9.2±2.2	9.5±2.3	3.381	0.223
**Negative self-image**	3	12	6.9±1.6	6.8, 7.0	57.5 (56.3, 58.7)	6.8±1.6	7.1±1.7	0.882	**0.026**
**Concern with public attitude**	10	40	24.4±4.9	24.0, 24.9	61.0 (60.0, 62.2)	24.2±4.9	24.7±5.0	1.809	0.298
**Total stigma score**	17	68	40.6±8.0	39.9, 41.3	59.7 (58.7, 60.7)	40.3±7.8	41.3±8.7	4.682	0.198

^a^ F statistic for Independent Samples t-test or χ2 statistic for Chi-squared test

^b^ P-values ≤ 0.05 indicate statistically significant differences between participants who were directly involved in the care of COVID-19 patients and those who were not.

In the bivariate analysis, direct involvement in the care of COVID-19 patients was significantly associated with a higher proportion of severe stigma level [54 (39.1%) versus 105 (28.3%), p = 0.050] and participants’ higher scores for the negative self-image subscale **Table 3**.

### COVID-19-related experiences

Most participants thought they should stay away from their families until COVID-19 subsides if they were in regular contact with COVID-19 patients *“associative stigma”* (n = 463, 91.0%) and felt guilty they might expose their families to infection *“self-stigma”* (n = 462, 90.8%). A quarter of the sample reported their neighbours were the people most stigmatizing them (n = 129, 25.3%), and almost a half of the participants were most stigmatized by others (not co-workers/ family/ friends/ neighbours/ household members) *“experienced discrimination”* (n = 251, 49.3%). However, less than a fifth of the participants thought they should hide a positive test result in case they had one *“anticipated stigma”* (n = 88, 17.3%) **Table 4**.

**Table 4:** COVID-19-related experiences and total COVID-19-related stigma scores among a sample of HCWs, Egypt, 2020 (n = 509).

	Total	Directly involved in the care of COVID-19 patients		Statistic^b^	p-value^c^	Total stigma score	Statistic^d^	p-value^e^
		No	Yes			Mean±SD		
	N = 509	n = 371	n = 138					
**Do you think if you are in regular contact with COVID-19 patients, you should stay away from your family till the crisis subsides?**								
No	46 (9.0)	23 (6.2)	23 (16.7)	13.406	**<0.001**	39.6±10.5	6.874	0.396
Yes	463 (91.0)	348 (93.8)	115 (83.3)			40.7±7.8		
**Do you think that you need to hide that you have a positive test result of COVID-19, in case that happened?**								
No	421 (82.7)	312 (84.1)	109 (79.0)	1.838	0.175	40.2±7.8	3.971	**0.020**
Yes	88 (17.3)	59 (15.9)	29 (21.0)			42.4±9.1		
**Do you feel guilty that you might potentially expose your family to infection with COVID-19? **								
No	47 (9.2)	34 (9.2)	13 (9.4)	0.008	0.929	35.5±9.5	2.200	**<0.001**
Yes	462 (90.8)	337 (90.8)	125 (90.6)			41.1±7.7		
**In your opinion, has the media played a role in addressing COVID-19 public stigma against HCWs? **								
No effect	134 (26.3)	105 (28.3)	29 (21.0)	5.149	0.076	38.6±8.7	10.769	**<0.001**
Increased stigma	329 (64.6)	229 (61.7)	100 (72.5)			41.8±7.5		
Decreased stigma	46 (9.0)	37 (10.0)	9 (6.5)			37.8±7.9		
**Who are the people most stigmatizing you? **								
Co-workers	40 (7.9)	30 (8.1)	10 (7.2)	1.584	0.903	41.7±5.6	5.856	**<0.001**
Family	64 (12.6)	47 (12.7)	17 (12.3)			41.2±8.7		
Friends	2 (0.4)	2 (0.5)	0			40.0±1.4		
Household members	23 (4.5)	15 (4.0)	8 (5.8)			37.9±9.2		
Neighbors	129 (25.3)	95 (25.6)	34 (24.6)			43.4±7.7		
Others	251 (49.3)	182 (49.1)	69 (50.0)			39.1±7.9		
**Do you think that people are excused to refuse burial of dead COVID-19 patients? **								
No	434 (85.3)	323 (87.1)	111 (80.4)	3.516	0.061	40.4±8.0	0.043	0.251
Yes	75 (14.7)	48 (12.9)	27 (19.6)			41.6±8.2		
**Have you received any psychological support/counselling to cope with the COVID-19 pandemic? **								
No	429 (84.3)	315 (84.9)	114 (82.6)	0.401	0.527	40.4±8.2	5.232	0.238
Yes	80 (15.7)	56 (15.1)	24 (17.4)			41.6±6.7		
**What were your major concerns during the COVID-19 epidemic?**^**a**^								
Health	434 (85.3)	321 (86.5)	113 (81.9	1.723	0.189	40.6±8.0	1.615	0.842
Financial	121 (23.8)	88 (23.7)	33 (23.9)	0.002	0.964	40.5±7.9	0.009	0.833
Social	110 (21.6)	78 (21.0)	32 (23.2)	0.278	0.598	42.1±7.2	1.751	**0.022**
Other	12 (2.4)	7 (1.9)	5 (3.6)	1.317	0.251	40.8±11.7	4.033	0.944
**What measures do you think would have helped you the most during this epidemic?**^**a**^								
Availability of online training/educational sessions	191 (37.5)	145 (39.1)	46 (33.3)	1.419	0.234	41.4±7.6	0.904	0.100
Orientation of the public through mass media	195 (38.3)	153 (41.2)	42 (30.4)	4.969	0.026	39.6±7.5	1.335	**0.024**
Provision of protective equipment	174 (34.2)	185 (49.9)	89 (64.5)	8.660	0.003	40.8±7.9	0.110	0.525
Other	45 (8.8)	27 (7.3)	18 (13.0)	4.149	0.053	40.9±9.4	1.776	0.764

a More than one option was allowed; numbers do not sum to total

b χ2 statistic for Chi-squared test

c P-values ≤ 0.05 indicate statistically significant differences between participants who were directly involved in the care of COVID-19 patients and those who were not.

d F statistic for Independent Samples t-test or ANOVA

e P-values ≤ 0.05 indicate statistically significant differences of total stigma scores by background characteristics

Most of the participants thought that media increased COVID-19 public stigma against HCWs (n = 329, 64.6%). During the COVID-19 pandemic, the most common concern reported by the participants was their health (n = 434, 85.3%) and the most helpful measure they suggested was orientation of the public through mass media (n = 195, 38.3%) **Table 4**.

Participants directly involved in the care of COVID-19 patients were significantly more likely to recommend provision of protective equipment as the most helpful measure during the COVID-19 pandemic than those who were not directly involved in the care of COVID-19 patients **Table 4**.

### Factors associated with COVID-19-related stigma

In the multivariable regression models, participants’ overall COVID-19-related stigma score was negatively associated with higher qualification (β = -0.19, 95% CI: 2.32, -0.64, p = 0.001), and positively associated with working in an quarantine hospital (β = 0.08, 95% CI: 0.01, 7.14, p = 0.050). For the four subscales, all were also negatively associated with higher qualification. In addition, negative self-image was negatively associated with increasing age and positively associated with working in a fever hospital. Concern with public attitudes was negatively associated with increasing age and positively associated with working in an quarantine hospital **Table 5**.

**Table 5:** Multivariable linear regression models for factors associated with COVID-19 related stigma and its subscales among a sample of HCWs, Egypt, 2020 (n = 509).

	Overall stigma score		Personalized stigma		Disclosure concerns		Negative Self Image		Concern with public attitude	
	β (95% CI)	p-value	β (95% CI)	p-value	β (95% CI)	p-value	β (95% CI)	p-value	β (95% CI)	p-value
Adjusted R2	0.091		0.073		0.068		0.084		0.104	
**Increasing age**	-0.09 (-1.51, 0.09)	0.083	-0.13 (-0.51, -0.08)	0.007	0.10 (-0.29, 0.75)	0.380	-0.15 (-0.43, -0.09)	**0.003**	-0.11 (-1.02, -0.04)	**0.035**
**Female**	0.05 (-0.73, 2.37)	0.300	-0.01 (-0.48, 0.38)	0.812	0.01 (-0.90, 0.97)	0.945	0.04 (-0.17, 0.44)	0.392	0.05 (-0.38, 1.52)	0.237
**Married**	0.03 (-1.29, 2.36)	0.568	NA		-0.02 (-1.19, 0.99)	0.853	0.03 (-0.25, 0.50)	0.502	0.02 (-0.90, 1.34)	0.697
**Higher qualification**	-0.19 (2.32, -0.64)	**0.001**	-0.14 (-0.53, -0.10)	**0.005**	-0.31 (-1.09, -0.12)	**0.014**	-0.13 (-0.38, -0.04)	**0.016**	-0.20 (-1.49, -0.47)	**<0.001**
**Living in Cairo**	NA		NA** **		NA		NA		NA	
**Specialty**										
Internal Medicine	-0.05 (-2.54, 1.08)	0.427	-0.02 (-0.58, 0.42)	0.748	-0.10 (-1.67, 0.72)	0.431	NA		-0.04 (-1.53, 0.69)	0.457
Surgery	NA		NA		NA		NA		NA	
Other	0.08 (-0.61, 3.45)	0.170	0.06 (-0.28, 0.85)	0.329	0.02 (-1.20, 1.43)	0.867	0.07 (-0.09, 0.58)	0.149	0.07 (-0.41, 2.08)	0.188
**Workplace**										
Quarantine hospital	0.08 (0.01, 7.14)	**0.050**	0.08 (-0.07, 1.91)	0.068	-0.01 (-2.21, 1.95)	0.904	0.06 (-0.24, 1.30)	0.175	0.10 (0.37, 4.74)	**0.022**
Fever hospital	NA		NA		NA		0.09 (0.07, 4.53)	**0.043**	NA	
Public hospital	0.06 (-0.79, 3.37)	0.224	0.05 (-0.28, 0.87)	0.317	0.03 (-1.26, 1.60)	0.814	0.02 (-0.32, 0.54)	0.626	0.06 (-0.48, 2.07)	0.219
Primary healthcare center	NA		NA		NA		NA		NA	
University hospital	-0.06 (-2.55, 0.48)	0.181	-0.03 (-0.57, 0.27)	0.474	-0.03 (-1.11, 0.81)	0.762	-0.07 (-0.55, 0.08)	0.138	-0.04 (-1.33, 0.52)	0.394
Private hospital/clinic	NA		NA		NA		NA		NA	
Other	NA		NA		NA		NA		NA	
**Directly involved in the care of COVID-19 patients**	NA		NA		NA		0.04 (-0.18, 0.51)	0.355	NA	
**Works in a COVID-19 affected healthcare facility**	NA		NA		NA		0.04 (-0.18, 0.46)	0.393	NA	
**Positive test result**	NA		NA		0.18 (-0.03, 2.30)	0.056	NA		NA	
**R2**	0.108		0.087		0.142		0.104		0.120	

NA: not included in the model because the variable did not show statistical significance in the bivariate linear regression model.

## Discussion

This study is one of the first attempts to explore COVID-19-related stigma among HCWs using a stigma-specific tool and to assess its associated factors. In this sample of Egyptian physicians, our preliminary findings suggest that most participants experienced some level of COVID-19-related stigmatization. Lower qualification, younger age, and working in an quarantine or fever hospital were the factors independently associated with COVID-19-related stigma in the multivariable analyses.

Disease-related stigmatization was previously reported for the Autoimmune Deficiency Syndrome [23], the Middle East Respiratory Syndrome [26], and SARS [17, 27]. Similarly, the current COVID-19 pandemic may have led to stigmatizing experiences and practices, such as social exclusion, discrimination, self-blaming and shame [28]. In this exploratory study, approximately one third of the participants reported a severe level of COVID-19-related stigma. During previous respiratory disease epidemics, such as the 2004 SARS epidemic, 22% of the studied HCWs reported stigma and rejection in their neighbourhood [22]. A similar proportion of participants in this study (25.3%) experienced discrimination from their neighbours.

In the current study, direct involvement in the care of COVID-19 patients was significantly associated with participants’ higher scores for the negative self-image subscale and a higher proportion of severe level of stigma in the bivariate analysis. Similarly, a previous study found an association between higher stigma scores, particularly scores related to the concern with public attitudes subscale, and general practitioners’ involvement in treating SARS [17]. However, direct involvement in the care of COVID-19 patients was not independently associated with COVID-19-related stigma in the multivariable regression analyses, suggesting that stigma was universally experienced in this sample of Egyptian physicians, regardless of whether the nature of their work at the workplace required direct involvement in COVID-19 care pathways.

Participants’ total COVID-19-related stigma score, concern with public attitudes and negative self-image subscale scores were positively associated with working in quarantine and fever hospitals. Working in these healthcare facilities, regardless of HCWs’ direct involvement in the COVID-19 care pathway, seems to have introduced the COVID-19-related stigma ‘marking’ to these HCWs. A recent review of psychological consequences related to COVID-19 in healthcare workers and quarantined people revealed that stigma was reported as well as several emotional disorders, such as stress, anxiety, depression, sleep disorders and frustration [29].

According to the calculated percentiles of COVID-19-related stigma subscales, participants reported relatively higher stigma related to the concern with public attitudes subscale and comparatively lower stigma related to the negative self-image subscale; both subscales were inversely associated with increasing age. These findings are consistent with previous literature from the SARS epidemic in Singapore, where younger age was found to be significantly associated with higher stigma subscales [17]. Also, participants’ total COVID-19-related stigma score and all subscale scores were inversely associated with higher qualification. This might be explained by the lack of experience of those physicians, which highlights the importance of psychological support and training especially to junior staff.

Because of the ambiguity around COVID-19 and its evolving nature several false beliefs and myths might arise in the community [2] and be propagated by the media, especially with the ‘infodemic’ associated with the COVID-19 pandemic [10]. Approximately two-thirds of participants thought that media had a negative role in increasing COVID-19 public stigma in agreement with other reports [30]. Participants in this study perceived higher stigma related to the concern with public attitudes subscale and suggested that public orientation about the role of HCWs during the epidemic would be a helpful measure that needs to be addressed through mass media.

COVID-19-related stigmatization was addressed in press in many countries, such as Egypt [21], India [31], Malawi [32], and Mexico [33]. It was also tackled in scientific journals in the form of letters to the Editor [34]. However, COVID-19-specific stigma research is lacking despite that stigma may influence health outcomes not only on the individual level, but also on the population-level, by affecting, worsening or interfering with social relationships, stress, psychological and behavioral responses [35]. Unfortunately, stigma experiences like discrimination may last for a long time, even after the epidemic subsides [36]. A review of infectious diseases stated that HCWs felt greater stigmatization than the general population and experienced more avoidance behaviors from the community [29]. Most participants in this study reported other stigma experiences as well, such as *self-stigma* as they felt guilty exposing their families to infection with COVID-19 and *associative stigma* as participants thought they should stay away from their family till the crisis subsides, if they were in regular contact with COVID-9 patients.

Only 14.7% of all participants in this study received psychological support or counselling to cope with the COVID-19 pandemic. This proportion seems insufficient considering that most participants reported some form of COVID-19-related stigmatization. Assessing people’s knowledge about the disease to help identify stigmatization, collaboration with community organizations to address stigma, and raising community awareness and skills-building training to fight stigma are among the practical strategies to diminish disease-related stigma during an epidemic [37]. Psychiatrists from 13 countries recommended developing multi-component, multi-level, long-term, and broad interventions that address COVID-19-related stigma, its drivers and facilitators using the ‘Health stigma and discrimination framework’ [8, 10]. However, more systematic research is required for a deeper understanding of the underlying complexities concerning COVID-19-related stigma among HCWs. COVID-19 stigma-focused interventions targeting HCWs need development and incorporation into the existing system, specifically psychological and public support for younger HCWs and those with lower qualifications.

### Strengths and limitations

This study is one of the first endeavours to measure COVID-19-related stigma among HCWs. It included a considerable number of physicians, who worked in different healthcare facilities in Egypt and were directly and indirectly involved in the COVID-19 care pathway. It also examined various aspects of COVID-19-related stigma. This exploratory study highlights the need for specific research and targeted interventions particularly addressing COVID-19-related stigmatization among HCWs.

However, findings of this exploratory study are preliminary and should be further investigated in-depth with more robust study designs, sampling methods, and validated COVID-19-specific tools. Some limitations may prevent the generalizability of these results. First, due to the lack of a specific tool to measure COVID-19-related stigma, we had to use a scale designed to measure other health-related stigma. Verma et al. adapted a stigma scale, which was originally developed to measure HIV-related stigma [23], to measure post-SARS stigmatization among HCWs [17]. Although SARS and COVID-19 are different in their indices and clinical profiles, they are both respiratory infectious diseases that have caused epidemics. Further studies should be carried out to validate a specific scale for COVID-19-related stigma among HCWs. Second, the scarcity of studies on stigmatization against HCWs during the COVID-19 pandemic did not allow comparison of the findings of this study with the extent, level, and factors associated with stigma among HCWs in other settings. Third, self-selection bias may have been introduced due to the convenience nature of the sampling technique used in this study, where stigmatized physicians were more likely to choose participating in this study. Fourth, the cross-sectional study design disallows drawing causal inferences between COVID-19-related stigma and its associated factors. Future studies are required to address these limitations to advance understanding and cover the existing gaps in knowledge on COVID-19-related stigma among HCWs.

## Conclusions

Findings suggest that a considerable proportion of Egyptian healthcare providers in this study experienced COVID-19-related stigmatization, mainly from their neighbours and others they interacted with in the community during the COVID-19 pandemic. This may explain why participants’ COVID-19-related stigmatization was particularly higher for concerns with public attitudes about HCWs. COVID-19-related stigmatization was universally experienced in this sample of Egyptian physicians, regardless of whether the nature of their work at the workplace required direct involvement in COVID-19 care pathways. Thus, public health education and raising community and media awareness about the importance of public support for HCWs are necessary to alleviate their perceived stigma. The overall COVID-19-related stigma score and its subscale scores were inversely associated with higher participants’ qualification, highlighting the need for providing targeted psychological support for the younger and inexperienced physicians.

## Supporting information

S1 Questionnaire (DOCX)

S1 Data set (XLSX)
