# Supplementary material for: COVID-19-related stigmatization among a sample of Egyptian healthcare workers
Source: PLoS One. 2020 Dec 18;15(12):e0244172. doi: 10.1371/journal.pone.0244172 (PMC7748273; doi:10.1371/journal.pone.0244172)
Supplement: S1 Questionnaire — (DOCX) [file pone.0244172.s001.docx]

**Items of stigma scale and corresponding subscales (see reference)***

|  | **Items** | **Subscale assignment** |
| --- | --- | --- |
| **1** | Telling someone I am a HCW is risky | 2, 4 |
| **2** | HCW are treated like outcasts | 4 |
| **3** | Being a HCW, I feel set apart and isolated from the rest of the world | 1, 3, 4 |
| **4** | I am very careful who I tell that I am a HCW | 2 |
| **5** | Some people who know I am a HCW have grown more distant | 1 |
| **6** | I worry about people discriminating against me | 2, 4 |
| **7** | Most people nowadays are uncomfortable around a HCW | 4 |
| **8** | I never feel the need to hide the fact that I am a HCW | 2 |
| **9** | I have been hurt by how people reacted to learning I am a HCW | 1 |
| **10** | I regret having told some people that I am a HCW | 1 |
| **11** | As a rule, telling others that I am a HCW has been a mistake | 1, 3, 4 |
| **12** | Some people avoid getting close to me once they know I am a HCW | 1, 4 |
| **13** | Some people close to me are afraid others will reject them if it becomes known that I am a HCW | 1 |
| **14** | People don't want me around their children once they know I am a HCW | 1, 4 |
| **15** | People have physically backed away from me when they learn I am a HCW | 1, 4 |
| **16** | I have stopped socializing with some people because of their reactions to my being a HCW | 1 |
| **17** | People seem afraid of me once they learn I am a HCW | 1, 3, 4 |

***Reference:** Verma S, Mythily S, Chan YH, Deslypere JP, Teo EK, Chong SA (2004). Post-SARS psychological morbidity and stigma among general practitioners and traditional Chinese medicine practitioners in Singapore. Ann Acad Med Singapore 33(6):743-748.

**Questionnaire**

**Stigmatization of COVID-19 among physicians’ survey**

We are a group of researchers from Faculty of Medicine, Ain Shams University. We are conducting a research study on the impact of COVID-19 epidemic on physicians in Egypt. It will take about 10 minutes to complete the questionnaire. We appreciate your time. We confirm that all data are anonymous, will be confidential and used for research purposes only.

I agree to participate in this research study

**Section 1: Personal and occupational data**

**Age:** ……

**Gender:** male female

**Marital status:**  Married Not married

**Qualification:** MBBCh Diploma Master Doctorate

**Specialty:**

[Allergy & Immunology](http://www.abai.org/)

[Anesthesiology](http://www.theaba.org/)

Chest

Clinical Pathology

[Dermatology](http://www.abderm.org/)

[Emergency Medicine](http://www.abem.org/)

ENT

[Family Medicine](http://www.theabfm.org/)

[Internal Medicine](http://www.abim.org/)

[Neurosurgery](http://www.abns.org/)

[Obstetrics & Gynecology](http://www.abog.org/)

[Ophthalmology](http://www.abop.org/)

[Orthopaedic Surgery](http://www.abos.org/)

[Pediatrics](http://abp.org/)

[Physical Medicine & Rehabilitation](http://www.abpmr.org/)

[Plastic Surgery](https://www.abplasticsurgery.org/)

[Psychiatry & Neurology](http://www.abpn.com/)

[Radiology](http://www.theabr.org/)

[Surgery](http://www.absurgery.org/)

[Thoracic Surgery](http://www.abts.org/)

[Urology](http://www.abu.org/)

other (specify)…..

**Work place** (check all that apply):

Primary healthcare centre

Public hospital

Private hospital/clinic

University hospital

Fever hospital

Chest hospital

Isolation hospital

**Are you directly involved in the care of COVID-19 patient (s)?**

Yes No

**Are you currently working in COVID-19 affected healthcare facility?**

Yes No

**If yes, In which Care pathway are you involved?**

Triage clinic Isolation rooms ICU

**Section 2: Stigma scale against Healthcare workers (HCW) (Reference: Verma S, Mythily S, Chan YH, Deslypere JP, Teo EK, Chong SA (2004). Post-SARS psychological morbidity and stigma among general practitioners and traditional Chinese medicine practitioners in Singapore. Ann Acad Med Singapore 33(6):743-748.)**

Strongly Disagree “SD”," Disagree “D”, Agree “A”, Strongly Agree “SA”

1. Telling someone I am a HCW is risky SD D A SA

2. HCW are treated like outcasts SD D A SA

3. Being a HCW, I feel set apart and

isolated from the rest of the world SD D A SA

4. I am very careful who I tell that I am a HCW SD D A SA

5. Some people who know I am a HCW have grown

more distant SD D A SA

6. I worry about people discriminating against me SD D A SA

7. Most people nowadays are uncomfortable around a HCW SD D A SA

8. I never feel the need to hide the fact that I am a HCW SD D A SA

Many of the items in this next section assume that you have told other people that you are a HCW, or that others know. This may not be true for you. If the item refers to something that has not actually happened to you, please imagine yourself in that situation. Then give your answer ("strongly disagree," "disagree," "agree," "strongly agree") based on how you think you would feel or how you think others would react to you.

9. I have been hurt by how people reacted to learning I am a HCW SD D A SA

10. I regret having told some people that I am a HCW …….SD D A SA

11 As a rule, telling others that I am a HCW has been a mistake ……. SD D A SA

12. Some people avoid touching me once they know I am a HCW….. SD D A SA

13. Some people close to me are afraid others will reject

them if it becomes known that I am a HCW ……. SD D A SA

14. People don't want me around their children once

they know I am a HCW SD D A SA

15. People have physically backed away from me when

they learn I am a HCW SD D A SA

16. I have stopped socializing with some people because

of their reactions to my being a HCW SD D A SA

17. People seem afraid of me once they learn I am a HCW SD D A SA

**Section 3: Stigma experiences**

**Do you think that if you are in regular contact with COVID-19 patients, you should stay away from your family till the crisis subsides?**

Yes No

**Do you think you need to hide that you have a positive test result of COVID-19, in case that happened?**

Yes No

**Do you feel guilty that you might potentially expose your family to infection with COVID-19?**

Yes No

**In your opinion, has the media (including TV, radio broadcasts, news, social media….) played a role in addressing COVID-19 public stigma against HCWs?**

Increased the stigma Decreased the stigma

**Who are the most stigmatizing you**?

Household members

Family

Neighbours

Co-workers

Others

**Do you agree with people refusing burial of dead COVID-19 patients?**

Yes No

**Have you personally done any tests to check whether you got infected with COVID-19?**

Yes No

**If yes, what was the test?**

PCR Antibodies

**If you were tested, what was the result?**

Positive Negative

**Have you received any psychological support/counselling to cope with COVID-19 pandemic?**

Yes No

**What were your major concerns during the COVID 19 epidemic?**

…………………………………….

**What measures do you think would have helped you the most during this epidemic?**

…………………………………….
